# A Multimodal Analysis of Serum and Tear Crystallization Patterns in Patients with Obesity

**DOI:** 10.3390/jcm15020773

**Published:** 2026-01-17

**Authors:** Cosmin Victor Ganea, Anisia Iuliana Alexa, Nicoleta Anton, Calina Anda Sandu, Madalina Ioana Bilha, Vlad Constantin Donica, Irina Andreea Pavel, Roxana Elena Ciuntu, Camelia Margareta Bogdanici

**Affiliations:** 1 Doctoral School, University of Medicine and Pharmacy “Grigore T. Popa”, University Street No. 16, 700115 Iasi, Romania; cosmin-victor.ganea@umfiasi.ro (C.V.G.); calina-anda.sandu@umfiasi.ro (C.A.S.); madalinabilha@gmail.com (M.I.B.); 2 Department of Ophthalmology, Faculty of Medicine, University of Medicine and Pharmacy “Grigore T. Popa”, University Street No. 16, 700115 Iasi, Romania; anton.nicoleta1@umfiasi.ro (N.A.); vlad-constantin.donica@umfiasi.ro (V.C.D.); andreea.niagu@umfiasi.ro (I.A.P.); roxana-elena.ciuntu@umfiasi.ro (R.E.C.); camelia.bogdanici@umfiasi.ro (C.M.B.)

**Keywords:** dry eye syndrome, diagnostic pattering test, tear biomarkers, serum biomarkers, obesity, body mass index, visceral fat

## Abstract

**Objectives**: The study examined tear and serum alterations using the ferning test and quantified the number of branches formed during the controlled drying of these biological fluids (tears and serum), in order to identify a potential diagnostic patterning test in individuals with obesity. **Methods**: A total of 61 patients aged between 25 and 72 years were enrolled (median age [interquartile range] = 39.0 [26] years). BMI values ranged from 19.1 to 47.5 kg/m^2^, with a median BMI (interquartile range) of 29.3 (12.1) kg/m^2^. **Results**: The Kruskal–Wallis test showed statistically significant differences among at least two Schirmer classes with respect to the number of branches observed in dried tears at a brightness threshold of 220 (H(2) = 8.485, *p* = 0.014). According to the Dunn post hoc test, Schirmer Class 1 showed a markedly lower number of branches compared with Classes 2 and 3 (*p* < 0.031 and *p* < 0.021), whereas no significant difference was found between Classes 2 and 3. The Kruskal–Wallis test further suggested the presence of statistically significant differences in the number of branches in dried serum, quantified using ImageJ2 at a brightness threshold of 190, across visceral fat classes (H(2) = 9.583, *p* = 0.008). Dunn’s post hoc tests revealed that the number of branches in serum analyzed at a brightness threshold of 190 was significantly higher in visceral fat class 3 compared to class 1 (*p*_holm_ = 0.006), while no statistically significant differences were observed between classes 1 and 2 or between classes 2 and 3 (*p*_holm_ > 0.05). **Conclusions**: In addition to other obesity-specific complications patients with obesity exhibit an increased risk of developing dry eye syndrome. The combined assessment of DPT in both the tear film and serum may represent a new method for analyzing obesity-associated biomarkers. Further studies are required to determine the sensitivity and specificity of these approaches in diagnosing systemic alterations induced by excess adipose tissue.

## 1. Introduction

Obesity is approaching epidemic proportions, with projections indicating that by 2035 approximately one-quarter of the global population will be affected [1]. The systemic inflammatory state associated with obesity is characterized by elevated plasma biomarkers, including C-reactive protein, interleukin-6, tumor necrosis factor-alpha, and leptin. This persistent, low-grade inflammation promotes the development of cardiovascular, endocrine, gastrointestinal, and ophthalmological diseases [2].

The tear ferning test and the examination of crystallization patterns in dried serum samples offer complementary perspectives on tear film changes and systemic alterations linked to obesity. Because tears and serum differ in both composition and physicochemical behavior, the resulting crystallization patterns should not be treated as the same phenomenon. Instead, they reflect fluid-specific responses that may capture different aspects of an individual’s metabolic state. Plasma constitutes the liquid component of blood, serving as the medium in which the formed elements—erythrocytes, leukocytes, and thrombocytes—are suspended. Moreover, plasma functions as the transport vehicle for oxidative by-products arising from protein, lipid, and carbohydrate metabolism [3,4].

Historically, the ferning test has served as a research tool for studying the characteristics of the tear film and the ways in which these properties are altered in various disease conditions. Diagnostic patterning tests (DPTs) represent an increasingly prominent methodology employed in contemporary studies to predict disease states [5,6].

The first meta-analysis to evaluate the role of DPT in cancer detection, “Multi-cancer Detection Using Pattern Formation in Drying Body Fluids: A Systematic Review and Meta-Analysis of Diagnostic Test Accuracy Studies” showed that despite substantial heterogeneity across included studies, DPT showed utility in detecting more than 50 types of malignancies [7].

DPT has been employed in the assessment of acute and chronic viral hepatitis, hepatic cirrhosis, oropharyngeal cancer, and bladder cancer. Such diagnostic patterns can be identified through the Serum Crystallization Patterns (SCPs). In patients with acute viral hepatitis, Botirov et al. described a distinctive branching configuration in desiccated serum, referred to as the “three-prong” pattern. Serum alterations associated with obesity likewise manifest as distinct crystallization patterns, which may possess the capacity to predict metabolic impairment induced by obesity [8,9].

The Tear Ferning Test (TFT) has been evaluated in patients with sarcoidosis-induced dry eye syndrome. The authors of the study reported that individuals with sarcoidosis exhibit a greater number of branches in the tear crystallization pattern compared to healthy subjects or to patients with dry eye syndrome of other etiologies [10].

The key purpose of this study was to explore how obesity might affect tear-film crystallization patterns as assessed by the Tear Ferning Test. Dry eye syndrome was evaluated as a clinically relevant sign of ocular surface involvement in individuals with excess body weight, with the Schirmer test used as the reference measure of tear production. Serum crystallization patterns were examined as an exploratory process to determine whether obesity might also influence systemic crystallization behavior. The DPT framework enabled a multimodal comparison between tear samples and serum patterns.

Based on this approach, we expected obesity to be linked to measurable changes in tear-ferning morphology, reflected in differences in branch number across Schirmer categories. We also anticipated that greater systemic adiposity would be associated with distinct serum crystallization patterns. In addition, we considered that alterations in the tear film and in serum crystallization might occur simultaneously, supporting the relevance of the DPT framework as a multimodal analytical tool in obesity research.

## 2. Materials and Methods

The study design and protocol were performed according to the tenets of the Declaration of Helsinki for research involving human subjects and approved by the Ethics Committee of “Grigore T. Popa” University of Medicine and Pharmacy Iasi, Romania (No. 663/approval date on 28 October 2025). Written informed consent was obtained prior to patient evaluation.

### 2.1. Subjects

A total of 61 individuals were selected, aged between 25 and 72 years (median age [interquartile range] = 39.0 [26] years). BMI values ranged from 19.1 to 47.5 kg/m^2^, with a median BMI (interquartile range) of 29.3 (12.1) kg/m^2^.

All patients met the study’s eligibility criteria, which required overnight fasting from both food and liquids to avoid interference with bioimpedance measurements, as well as the absence of cosmetic products to prevent potential errors during tear collection. Additionally, only naive patients were selected—those without prior ophthalmological examinations, without a history of ophthalmic surgery, and without topical ocular treatments during the preceding month. All patients with obesity enrolled in the study reported no known associated pathologies and no chronic medication use at home.

### 2.2. BMI

Patients were categorized according to BMI into the following groups: normal weight (20–24.9 kg/m^2^), overweight (25–29.9 kg/m^2^), and obesity (≥30 kg/m^2^). Body composition analysis was conducted using a Tanita BC-545N Digital scale (Tokyo, Japan), and stature was measured with a Seca 206 stadiometer.

### 2.3. Schirmer Test

Tear production was measured using the Schirmer I test, carried out without topical anesthesia and following standard clinical procedures. The filter paper strip was folded at the 5 mm mark and positioned in the lateral canthus to avoid corneal injury. Patients were instructed to keep their eyes closed for five minutes, after which the extent of paper wetting was quantified according to the graduated scale printed on the Schirmer strip.

None of the study participants exhibited severe dry eye disease, defined as a Schirmer value below 5 mm. Accordingly, patients were classified into three categories: Class 1 (moderate dry eye, corresponding to values of 5–9 mm), Class 2 (mild dry eye, corresponding to values of 10–14 mm), and Class 3 (normal tear production, defined by values ≥ 15 mm).

### 2.4. Tear Ferning Test

A known tear volume of approximately 3 μL was aspirated using the tip of a 10 μL micropipette. Tears were collected from the lateral canthus to remove any risk of corneal injury and were then deposited onto a glass slide for drying under controlled environmental conditions (24 °C, 46% humidity). The slides were examined using a Levenhuk MED D45T Digital Trinocular phase-contrast microscope (Levenhuk, Tampa, FL, USA). Images were captured through the primary lens of an iPhone 15 Pro Max (Apple Inc., Cupertino, CA, USA) attached to the microscope via an ocular adaptor.

For the quantitative analysis, a branch was defined as a single crystallization element identified in the binary-transformed image after threshold-based segmentation. The term “number of branches” refers to the total count of these discrete crystallization structures within the selected region of interest, using a fixed brightness threshold (220 for tear samples and 190 for serum). These values were generated using the particle analysis function in ImageJ2.

### 2.5. Serum Crystallization Patterns

A volume of 10 mL of venous blood was collected into MASTERLAB clot activator vacutainers (6 mL) (Conversano, Italy) and allowed to clot for 25 min. The samples were subsequently centrifuged using a Hettich centrifuge (model D-78532, Tuttlingen, Germany) for 15 min at 35,000 revolutions per minute. The resulting serum was aspirated with a 5 mL syringe and transferred into Eppendorf tubes. Using the tip of a 10 μL micropipette, a precisely measured 3 μL aliquot was then deposited onto a glass slide. The serum droplets were allowed to desiccate for 25 min.

### 2.6. Methodological Considerations for Reproducibility

Tear samples were allowed to dry for 10 min under controlled conditions (24 °C and 46% humidity), leading to well-defined crystallization patterns without peripheral artifacts. Serum samples needed a longer drying time of 25 min to develop well-defined crystallization branches. Image analysis was performed in ImageJ2 by adjusting only the brightness level (set to 220 for tear samples and 190 for serum), with saturation and hue left unchanged. Once the crystallization branches were identified using brightness thresholds, the images were converted to binary format, allowing objective branch counting through automated particle analysis. This standardized workflow was used consistently for all samples to minimize operator-dependent variability and improve reproducibility.

### 2.7. Visceral Fat Classification

Visceral fat class was determined using bioelectrical impedance analysis performed with a Tanita BC-545N Digital scale (Tanita, Tokyo, Japan). The device generates a visceral fat score derived from a proprietary algorithm. Based on the obtained values, patients were assigned to one of three visceral fat classes: Class 1 (normal range, scores 1–9), Class 2 (high risk, scores 10–14), and Class 3 (very high risk, scores ≥ 15).

### 2.8. Statistical Analysis

The data were processed and analyzed using the statistical software JASP Team (2024), JASP (Version 0.19.2) [Computer software], with the significance threshold set at 95%. A *p*-value < 0.05 was considered indicative of statistical significance.

A total of 61 individuals aged between 25 and 72 years were evaluated (median age [interquartile range] = 39.0 [26] years), exhibiting a non-normal distribution of values (Shapiro–Wilk *p* < 0.001). BMI values ranged from 19.1 to 47.5 kg/m^2^ and likewise showed a non-normal distribution (Shapiro–Wilk *p* = 0.010); therefore, the median BMI of 29.3 (12.1) kg/m^2^ was used in subsequent analyses.

Numerical variables were assessed for normality using the Shapiro–Wilk test. Variables conforming to a normal distribution were expressed as mean ± standard deviation, whereas non-normally distributed variables were reported as median and interquartile range (IQR).

For comparisons involving more than two independent groups with non-normally distributed data, the Kruskal–Wallis test was employed. The rank η^2^ reported by this test represents a nonparametric measure of effect size, distinct from the classical η^2^ coefficient in that it is calculated based on ranked rather than raw data. Although the Kruskal–Wallis test was selected as the primary statistical method-given the deviations from normality shown by the Shapiro–Wilk test—one-way analysis of variance (ANOVA) was applied as a complementary procedure to further assess the robustness of the findings.

The authors used OpenAI (ChatGPT 5.2) to assist in the interpretation of statistical results by comparing AI-generated interpretations with the authors’ own analysis. The final interpretation, conclusions and responsibility for the content rest entirely with the authors.

## 3. Results

The images were processed using the ImageJ2 analysis software. Tear ferning patterns were evaluated at a brightness threshold of 220, while SCP were assessed at a brightness threshold of 190.

To enhance the reproducibility of the analysis, the number of branches was calculated at multiple brightness levels, evaluated across sequential 5-unit intervals. The brightness values at which the differences between two adjacent thresholds approached zero were determined to be 220 for tear analysis and 190 for serum analysis.

Descriptive statistics for the study sample are presented in Table 1.

**Figure 1:**
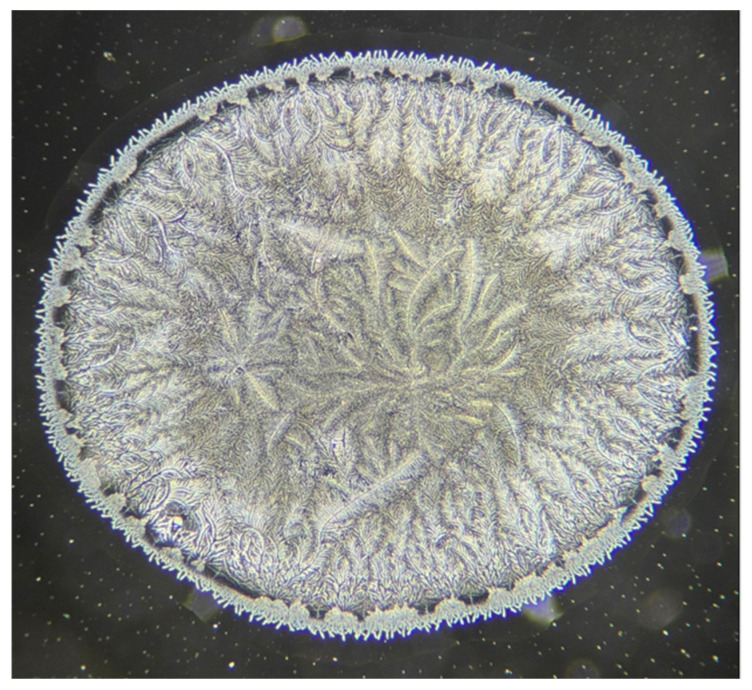
Phase-contrast micrograph of a dried 3 μL tear sample examined under ambient conditions. Images were acquired with a Levenhuk MED D45T trinocular phase-contrast microscope using a 10× phase-contrast objective. At this magnification, the field of view highlights the central crystallization branches used for quantitative analysis, while the peripheral evaporation zone is only partially represented. The dried droplet has an approximate diameter of 1720 μm.

**Figure 2:**
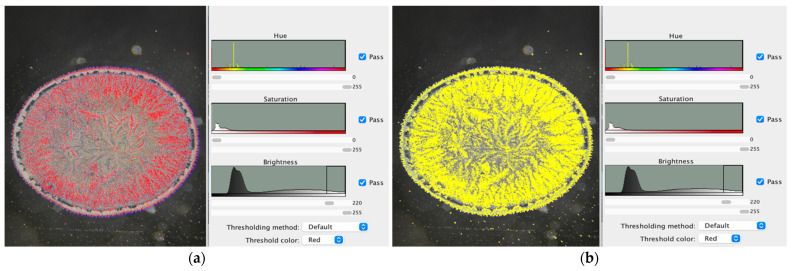
(**a**) Brightness adjustment; (**b**) Selection of the tear crystallization model.

**Figure 3:**
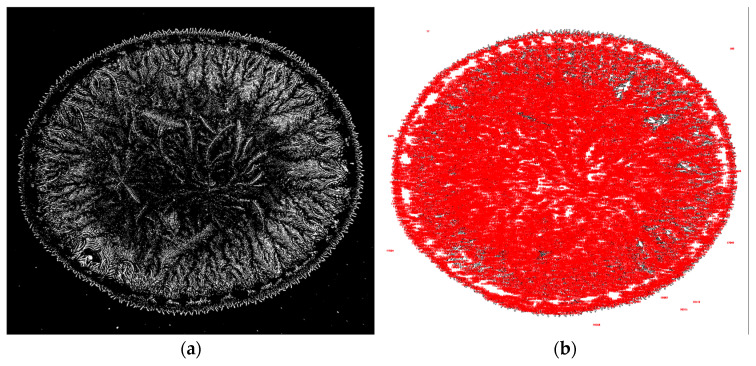
(**a**) Binary transformation; (**b**) Quantification of tear-fern branches using ImageJ2.

**Figure 4:**
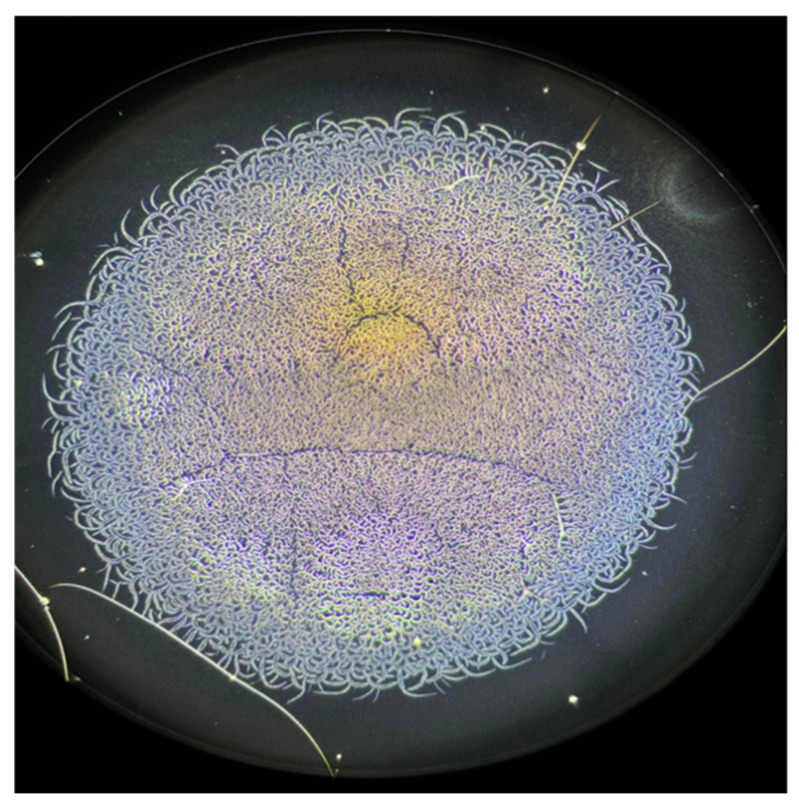
Phase-contrast micrograph of a dried 3 μL serum sample examined under ambient conditions. The images were captured with a Levenhuk MED D45T trinocular phase-contrast microscope using a 10× phase-contrast objective. At this magnification, the central region and the main crystallization branches—those used for quantitative analysis—are clearly visible, while the peripheral evaporation zone is only partially represented. The dried droplet measures approximately 1720 μm in diameter.

**Figure 5:**
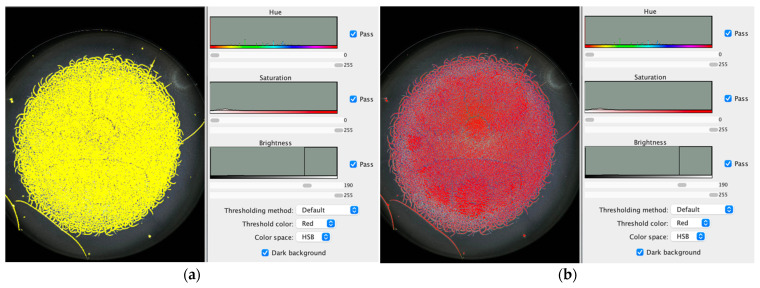
(**a**) Brightness adjustment; (**b**) Selection of the serum crystallization patterns.

**Figure 6:**
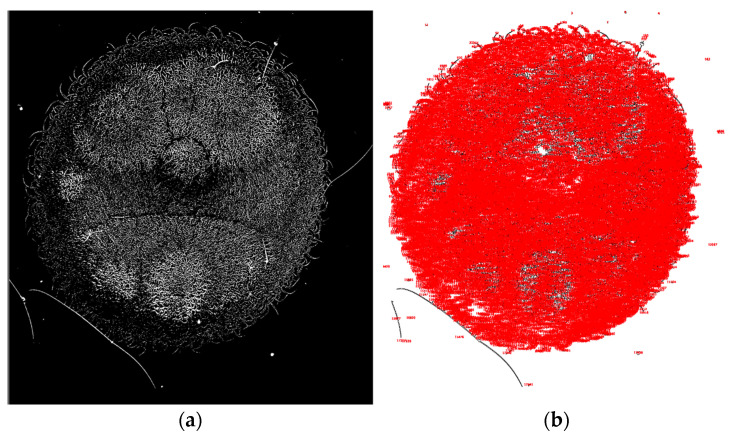
(**a**) Binary transformation; (**b**) Quantification of serum crystallization patterns using ImageJ2.

Figure 1 illustrates the dried tear sample selected for analysis. Figure 2 shows the adjustment of brightness intensity used to isolate the crystallization ferns formed during the drying process of biological fluids (tears). Figure 3 displays the application of binary transformation and particle analysis required for quantifying branches in dried tear samples. Figure 4 depicts the micelle-like appearance characteristic of dried plasma. Figure 5 shows the settings used to identify branches in desiccated serum. Figure 6 presents the binary transformation and the quantified number of serum branches.

**Figure 7:**
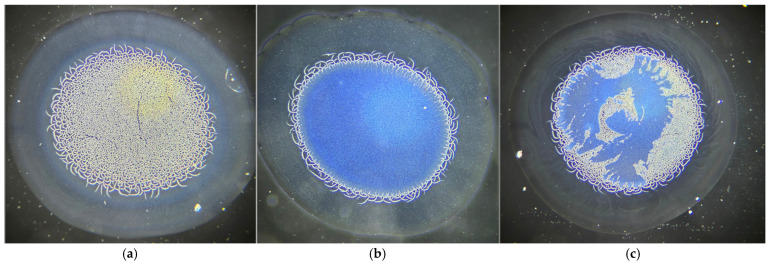
(**a**) This configuration was observed predominantly in normal-weight individuals; (**b**) In addition, among normal-weight patients, a classical ferning pattern—resembling that of dried tears—was identified along the surface of the micelle-like exoskeleton; (**c**) A third crystallization pattern emerged in patients with obesity, characterized by a “moth-eaten” appearance representing a hybrid of the two previously described crystallization models.

The serum samples exhibited a complex micelle-like crystallization pattern (Figure 7).

To enhance the reproducibility of the analytical procedure, ImageJ2 was employed to quantify the branches resulting from the crystallization of dried tears. We subsequently investigated the statistical relationship between tear samples analyzed at a brightness threshold of 220 and the corresponding Schirmer classification. It is important to note that none of the participants exhibited a Schirmer value below 5 mm, which would show severe dry eye disease. The Schirmer test results were therefore categorized as follows: Class 1—moderate dry eye (5–9 mm), Class 2—mild dry eye (10–14 mm), and Class 3—normal tear production (≥15 mm).

Although the one-way ANOVA initially appeared to show statistical significance (*p* = 0.017), this result must be interpreted with caution given the nonparametric distribution of the Schirmer values (Shapiro–Wilk *p* < 0.001). Consequently, the Kruskal–Wallis test was applied, as it does not assume normality and is more appropriate for ordinal variables such as the millimeter-based Schirmer classification (Table 2, Table 3 and Table 4).

The results of the Kruskal–Wallis test show the presence of statistically significant differences among at least two Schirmer classes (H(2) = 8.485, *p* = 0.014). Only 11% of the variability in branches number is explained by the Schirmer classification, corresponding to a small-to-moderate effect size (rank η^2^ = 0.112) (Table 5).

Schirmer Class 1 exhibited a significantly lower number of branches compared with Classes 2 and 3, as quantified by the tear ferning test. No statistically significant differences were observed between Classes 2 and 3 (Table 6).

We further examined whether analogous changes could also be detected in dried serum samples through quantitative analysis of SCP.

In the scientific literature, diagnostic patterning tests (DPTs) are employed to identify various conditions based on the crystallization patterns of biological fluids. In this context, we observed a preferential arrangement of branches in desiccated serum. A nonparametric ANOVA approach was used, given the non-normal distribution of the serum brightness 190 variable (*p* < 0.001). Moreover, because visceral fat class is organized ordinally, the Kruskal–Wallis test was deemed the most appropriate analytical method (Table 7).

The median branches counts of serum analyzed at a brightness threshold of 190 exhibit an upward trend correlated with visceral fat class (Table 8).

The variances were not equal, thereby further justifying the use of the Kruskal–Wallis test for statistical evaluation (Table 9).

The Kruskal–Wallis test shows a statistically significant association between the number of branches in dried serum—quantified using ImageJ2 at a brightness threshold of 190—and visceral fat class (H(2) = 9.583, *p* = 0.008). Approximately 13% of the variability in branches number is explained by visceral fat classification, corresponding to a moderate effect size (rank η^2^ = 0.131) (Table 10).

Dunn’s post hoc tests revealed that the number of branches in serum analyzed at a brightness threshold of 190 was significantly higher in visceral fat Class 3 compared with Class 1 (*p*_holm_ = 0.006). No statistically significant differences were observed between Classes 1 and 2 or between Classes 2 and 3 (*p*_holm_ > 0.05) (Table 11).

## 4. Discussion

The article “Assessment of Tear Film in Subjects with a High Body Mass Index” was the first to employ the ferning test to investigate the impact of obesity on the tear film [11].

Subsequently, Masmali et al., through “Application of a New Grading Scale for Tear Ferning in Non-Dry Eye and Dry Eye Subjects”, advanced the methodology by introducing a five-grade classification system for tear ferning. This new scale replaced the earlier four-grade Rolando classification, offering improved precision and ease of use [12].

Regarding the tear ferning test (TFT), several studies have examined the influence of external factors on tear drying dynamics, such as “Repeatability and Diurnal Variation of the Tear Ferning Test”. By contrast, SCP conditions remain largely unstandardized—a limitation also emphasized in the systematic review “Multi-cancer Detection Using Pattern Formation in Drying Body Fluids: A Systematic Review and Meta-Analysis of Diagnostic Test Accuracy Studies”. Parameters such as ambient temperature and humidity must be kept constant to avoid artefactual variation in drying patterns. In the present study, all samples were analyzed at 24 °C and 46% humidity [7,13].

Blood samples were allowed to clot for 25 min, followed by centrifugation for 15 min at 35,000 rpm. The resulting serum was aspirated using a 5 mL syringe and stored in Eppendorf tubes. A standardized 3 μL volume was then placed onto a glass slide for drying over 25 min. Notably, the drying time required for serum was more than double that for tears, the latter requiring only 10 min [14,15].

Obesity-induced dry eye syndrome is often overlooked among the potential contributors to ocular surface disease [16]. In our group, higher adiposity was linked to altered tear crystallization patterns and lower tear production, as reflected by the Schirmer categories. Although inflammatory pathways were not measured directly, these findings are consistent with previous work suggesting that metabolic status can affect ocular surface health.

To enhance reproducibility, we preferred quantifying ferning patterns from dried tears and comparing these with Schirmer test outcomes. Schirmer Class 1 showed a clearly lower branch count than Classes 2 and 3, while Classes 2 and 3 did not differ significantly from each other. The reduced number of branches seen in participants with lower Schirmer scores likely reflects alterations in both the amount and quality of the tear film. Although Meibomian gland function was not assessed in this study, earlier research has associated obesity-related metabolic and inflammatory changes with Meibomian gland dysfunction and lipid layer instability. Therefore, while our findings do not directly confirm this mechanism, they are consistent with the idea that Meibomian gland alterations may contribute to the tear film disturbances observed in obesity [17,18].

Although rank-based effect sizes were used to estimate the strength of the associations observed, they should be viewed primarily in a statistical and exploratory context. The small to moderate effect sizes identified in this study suggest measurable, but not conclusive, relationships between crystallization patterns, tear production, and adiposity-related measures. At this stage, these values should not be interpreted as direct indicators of clinical impact; instead, they offer quantitative clues that crystallization-based pattern analysis may have biological relevance. Further studies linking these metrics to established clinical outcomes and biochemical markers will be necessary to determine their actual clinical significance. The clinical relevance of crystallization patterns and DPT methodology arises from their potential to detect early-stage diseases for which no established screening protocols exist, such as bladder cancer or oropharyngeal malignancies [19,20].

The application of the SCP for investigating obesity status shows a new methodological approach; to our knowledge, no references addressing this topic are currently available in the scientific literature. The visceral fat class shows a statistically significant correlation with the number of branches observed in dried serum. The relevance of this relationship is supported by the fact that abdominal visceral adipose tissue is metabolically active and contributes to the secretion of pro-inflammatory adipokines. The interconnections between lipid secretion and oxidation underlie the accumulation of fat within hepatic tissue. Consequently, chronic inflammation in obesity, together with acquired insulin resistance, leads to the development of non-alcoholic fatty liver disease (NAFLD) [2].

Recent large-scale studies indicate that obesity plays a key role in multisystem disease, driven by interconnected metabolic and inflammatory pathways that underscore its wide-ranging systemic impact [21]. Accordingly, clinical research has also identified connections between dry eye syndrome and cardiovascular disease, suggesting that ocular surface changes may mirror broader systemic processes [22].

The first article to examine correlations between plasma and tear cytokines—“Distinct Cytokine Profiles in Plasma and Tears Highlight Ophthalmologic Inflammation in Type 2 Diabetes Without Retinopathy”—showed no correspondence between plasma and tear cytokine concentrations in diabetic patients. Whether this finding extends to individuals with obesity remains unknown and represents a key direction for future research [23].

The same author further showed in “Differential Ophthalmological Profile in Patients with Coronary Artery Disease Coexisting with Type 2 Diabetes Mellitus” the presence of a distinctive inflammatory tear-cytokine profile in type 2 diabetes [24]. The use of tear film as a biomarker of systemic health is of increasing interest, given its non-invasive collection and high potential for population-scale screening. Several ongoing studies aim to diagnose diseases such as Alzheimer’s disease, Parkinson’s disease, multiple sclerosis, cystic fibrosis, and rheumatoid arthritis through tear analysis [25,26].

One of the limitations of studies conducted over the years has been their focus on quantifying a single biomarker in tears. This shortcoming was addressed in the Dry Eye Assessment and Management (DREAM) study, which analyzed individualized inflammatory profiles in patients with dry eye syndrome [27].

It is highly probable that, in the coming years, we will witness a revolution in the screening of systemic diseases through the analysis of biofluids. Each pathology or medical procedure is expected to correspond to a distinct cytokine profile, a concept already explored in the context of post-cataract surgery, post-intravitreous injection, and post-vitrectomy inflammatory responses [28,29].

Unlike the qualitative grading systems introduced by Rolando and Masmali—which rely heavily on subjective visual assessment—our study applies a quantitative, image-based approach that uses objective branch counting to improve both reproducibility and sensitivity.

Although obesity-related inflammation, adipokine imbalance, and metabolic liver involvement are well documented in the literature, we did not directly measure inflammatory markers, adipokine profiles, or hepatic parameters in this study. Consequently, any reference to these mechanisms should be understood as contextual and hypothesis-generating rather than as conclusions supported by our data. The crystallization differences observed here may reflect systemic metabolic changes associated with obesity, but the study design does not allow us to infer causality.

A further limitation of this study is the modest sample size and the small number of participants within certain subgroups after stratification by BMI, Schirmer class, and visceral fat grade. This imbalance may have affected the strength of the associations identified. To minimize this issue, we employed non-parametric statistical methods, which are more appropriate for small or unevenly distributed samples. Nonetheless, the findings should be interpreted with caution and considered exploratory, providing groundwork for future studies with larger, more balanced groups.

Furthermore, unlike the tear film—where recent studies have proposed structured analytic models—we did not identify a standardized method for the analysis of dried serum in the existing literature. Monthly hormonal fluctuations in the female participants may also have influenced the results. Additionally, lifestyle factors and dietary habits can induce alterations in tear crystallization patterns, with potential implications at the serum level as well.

Dry eye syndrome is a multifactorial condition involving shifts in both the quantity and quality of the tear film, along with underlying inflammatory processes. In this study, the Schirmer test was not used as an independent diagnostic measure; instead, it served as a reference for tear production and was interpreted alongside the quantitative crystallization patterns resulted from the tear ferning test. Our conclusion that individuals with obesity are at greater risk for dry eye is therefore based on the combined pattern of reduced tear-production categories and consistent microstructural alterations in tear crystallization, rather than on Schirmer values alone.

## 5. Conclusions

Patients with obesity exhibit an elevated risk of developing dry eye syndrome, in addition to other obesity-specific complications. The combined application of DPT to both the tear film and serum may become a new analytical approach for identifying biomarkers associated with obesity. Further studies are required to determine the sensitivity and specificity of these methods in diagnosing systemic alterations induced by excess adipose tissue.

## Figures and Tables

**Table 1:** Descriptive statistics of the study group according to BMI.

	Normal Weight (BMI 20–24.9 kg/m^2^)	Overweight (BMI 25–29.9 kg/m^2^)	Obesity (BMI > 30 kg/m^2^)
Number of patients	(n = 20)	(n = 14)	(n = 27)
Serum brightness 190	354 (680) branches	281 (437.5) branches	978 (3826) branches
Tear brightness 220	11,228.3 ± 4455.2 branches	11,312.7 ± 3794.5 branches	11,415.2 ± 3911.4 branches
Schirmer test	22.5 (26.2) mm	19.5 (23.2) mm	15 (24.5) mm
Visceral fat score	6 (3.5)	11 (2.7)	19 (7)
Age	28.5 (8) years	41 (19.5) years	53 (20.5) years

**Table 2:** ANOVA—tear brightness 220.

								95% CI for η^2^	
Homogeneity Correction	Cases	Sum of Squares	df	Mean Square	F	*p*	η^2^	Lower	Upper
None	Schirmer class	1.258 × 10^8^	2.000	6.288 × 10^7^	4.358	0.017	0.131	0.004	0.288
	Residuals	8.367 × 10^8^	58.000	1.443 × 10^7^					
Brown–Forsythe	Schirmer class	1.258 × 10^8^	2.000	6.288 × 10^7^	4.759	0.016	0.131	0.004	0.288
	Residuals	8.367 × 10^8^	31.842	2.628 × 10^7^					
Welch	Schirmer class	1.258 × 10^8^	2.000	6.288 × 10^7^	6.790	0.004	0.131	0.004	0.288
	Residuals	8.367 × 10^8^	26.801	3.122 × 10^7^					

Note. Type III Sum of Squares.

**Table 3:** Descriptives—tear brightness 220.

Schirmer Class	N	Mean	SD	SE	Coefficient of Variation
1	15	8858.667	2482.427	640.960	0.280
2	12	12,646.333	4226.327	1220.035	0.334
3	34	11,956.500	4097.258	702.674	0.343

**Table 4:** Test for Equality of Variances (Levene’s).

F	df1	df2	*P*
3.024	2.000	58.000	0.056

**Table 5:** Kruskal–Wallis Test.

					95% CI for Rank η^2^	
Factor	Statistic	df	*p*	Rank η^2^	Lower	Upper
Schirmer class	8.485	2	0.014	0.112	0.015	0.289

**Table 6:** Dunn’s Post Hoc Comparisons—Schirmer class.

Comparison	z	W_i_	W_j_	r_rb_	*p*	*p*_bonf_	*p*_holm_
1–2	−2.417	19.467	36.083	0.578	0.016 *	0.047 *	0.031 *
1–3	−2.695	19.467	34.294	0.475	0.007 **	0.021 *	0.021 *
2–3	0.300	36.083	34.294	0.044	0.764	1.000	0.764

Note. Rank-biserial correlation based on individual Mann–Whitney tests. * *p* < 0.05, ** *p* < 0.01.

**Table 7:** ANOVA—serum brightness 190.

								95% CI for η^2^	
Homogeneity Correction	Cases	Sum of Squares	df	Mean Square	F	*p*	η^2^	Lower	Upper
None	visceral fat class	4.476 × 10^7^	2.000	2.238 × 10^7^	4.139	0.021	0.125	0.002	0.281
	Residuals	3.136 × 10^8^	58.000	5.407 × 10^6^					
Brown–Forsythe	visceral fat class	4.476 × 10^7^	2.000	2.238 × 10^7^	3.605	0.037	0.125	0.002	0.281
	Residuals	3.136 × 10^8^	38.235	8.202 × 10^6^					
Welch	visceral fat class	4.476 × 10^7^	2.000	2.238 × 10^7^	4.870	0.016	0.125	0.002	0.281
	Residuals	3.136 × 10^8^	26.895	1.166 × 10^7^					

Note. Type III Sum of Squares.

**Table 8:** Descriptives—serum brightness 190.

Visceral Fat Class	N	Median	IQR	*p* Value of Shapiro-WIlk
1	25	299	570	<0.001
2	17	722	2330	<0.001
3	19	978	3767	<0.001

**Table 9:** Test for Equality of Variances (Levene’s).

F	df1	df2	*p*
8.638	2.000	58.000	<0.001

**Table 10:** Kruskal–Wallis Test.

					95% CI for Rank η^2^	
Factor	Statistic	df	*p*	Rank η^2^	Lower	Upper
visceral fat class	9.583	2	0.008	0.131	0.008	0.316

**Table 11:** Dunn’s Post Hoc Comparisons—visceral fat class.

Comparison	z	W_i_	W_j_	r_rb_	*p*	*p*_bonf_	*p*_holm_
1–2	−1.552	23.400	32.059	0.275	0.121	0.362	0.242
1–3	−3.082	23.400	40.053	0.554	0.002	0.006	0.006
2–3	−1.349	32.059	40.053	0.251	0.177	0.532	0.242

Note. Rank-biserial correlation based on individual Mann–Whitney tests.

## Data Availability

The original contributions presented in this study are included in the article. Further inquiries can be directed to the corresponding author.

## Abbreviations

The following abbreviations are used in this manuscript:

ANOVA	Analysis of variance
BMI	Body mass index
DPT	Diagnostic patterning test
IQR	Interquartile range
SCP	Serum crystallization patterns
TFT	Tear ferning test
